# Cytokines and Cytokine Receptors Involved in the Pathogenesis of Alzheimer’s Disease

**DOI:** 10.4172/2155-9899.1000441

**Published:** 2016-08-04

**Authors:** Tomone Nagae, Kiho Araki, Yuki Shimoda, Lucia I. Sue, Thomas G. Beach, Yoshihiro Konishi

**Affiliations:** 1 Department of Clinical Research, National Tottori Medical Center, Tottori 689-0203, Japan; 2 Civin Laboratory for Neuropathology, Banner Sun Health Research Institute, Sun City, AZ, 85351, USA

**Keywords:** Cytokines, Neuroinflammation, Alzheimer’s disease, Postmortem brain tissues, Tumor necrosis factor (TNF)α, TNF receptors

## Abstract

Inflammatory mechanisms are implicated in the pathology of Alzheimer’s disease (AD). However, it is unclear whether inflammatory alterations are a cause or consequence of neurodegeneration leading to dementia. Clarifying this issue would provide valuable insight into the early diagnosis and therapeutic management of AD. To address this, we compared the mRNA expression profiles of cytokines in the brains of AD patients with “non-demented individuals with AD pathology” and non-demented healthy control (ND) individuals. “Non-demented individuals with AD pathology” are referred to as high pathology control (HPC) individuals that are considered an intermediate subset between AD and ND. HPC represents a transition between normal aging and early stage of AD, and therefore, is useful for determining whether neuroinflammation is a cause or consequence of AD pathology. We observed that immunological conditions that produce cytokines in the HPC brain were more representative of ND than AD. To validate these result, we investigated the expression of inflammatory mediators at the protein level in postmortem brain tissues. We examined the protein expression of tumor necrosis factor (TNF)α and its receptors (TNFRs) in the brains of AD, HPC, and ND individuals. We found differences in soluble TNFα and TNFRs expression between AD and ND groups and between AD and HPC groups. Expression in the temporal cortex was lower in the AD brains than HPC and ND. Our findings indicate that alterations in immunological conditions involving TNFR-mediated signaling are not the primary events initiating AD pathology, such as amyloid plaques and tangle formation. These may be early events occurring along with synaptic and neuronal changes or later events caused by these changes. In this review, we emphasize that elucidating the temporal expression of TNFα signaling molecules during AD is important to understand the selective tuning of these pathways required to develop effective therapeutic strategies for AD.

## Introduction

### Are inflammatory alterations a cause or consequence of AD neurodegeneration?

The molecular mechanisms underlying Alzheimer’s disease (AD) pathogenesis have not yet been elucidated. Numerous inflammatory mediators have been identified in AD brains but were not detected in non-demented elderly control individuals. This suggests that various inflammatory mechanisms are involved in the process of AD [1–3]. An important role for neuroinflammation in AD pathogenesis is supported by findings that immune receptor genes, including the triggering receptor expressed on myeloid cells 2 (TREM2) and CD33, are associated with AD [4]. Changes in cytokine expression in AD are not restricted to a single cytokine; there are widespread changes in intracellular cytokine signaling networks [5]. Amyloid β (Aβ) deposits, neurofibrillary tangles (NFTs), and neuronal degeneration are characteristic of AD and are the most likely sources of inflammation in AD brains [3,4,6]. In contrast, Aβ and Aβ precursor protein (APP) are regulated by cytokines [3,7]. In addition, systemic and peripheral inflammation in the preclinical stage of AD may upregulate neuroinflammation, causing neuronal damage in AD [6,8–10]. The role of cytokines in the pathogenesis of AD beyond their relationship with Aβ has begun to attract attention [7]. Neuroinflammation is now thought to contribute to AD pathogenesis rather than being a consequence of emerging senile plaques, NFTs, and neurodegeneration [4,6]; however, this has not been confirmed [2,4,11,12]. Recent clinical trials for AD treatment based on the amyloid hypothesis have been disappointing, and drug development needs to address the multifactoriality of the disease [13–15]. AD pathogenesis involves neuroinflammation, which includes the production of neurotoxic and neurotrophic cytokines [4–6,8]. Our major focus has been to clarify whether neuroinflammation is involved in AD pathogenesis, particularly prior to the onset of overt AD dementia. If peripheral and neural alterations in inflammatory mediators are primary AD pathologic processes that occur before neuronal damage and dementia, then these alterations will provide valuable insights into early diagnosis and therapeutic management of AD [1,16]. Alternatively, the alterations may be secondary responses to AD, occurring in the later symptomatic stages of the disease.

Anti-inflammatory therapies, which are described in detail below, using non-steroidal anti-inflammatory drugs (NSAIDs) should prevent cognitive impairment in AD if inflammatory and immunological alterations precede AD dementia. Increasing reports have demonstrated alterations in the expression of inflammatory mediators in peripheral blood mononuclear cells, cerebrospinal fluid (CSF), plasma, and serum of mild cognitive impairment (MCI) individuals [17–23]. MCI is a preclinical stage of AD and represents a transitional period between normal aging and early AD [24]. Recently, Rodriguez-Vieitez et al. [16] detected astrocytic activation by positron emission tomography imaging with ^11^C-deuterium-L-deprenyl (^11^C-DED) in asymptomatic carriers of autosomal dominant AD with increasing Aβ-plaque load during disease progression. Such alterations in MCI and asymptomatic carriers of AD suggest that inflammatory events may precede the clinical development of AD [21,23] and that NSAIDs may interrupt or slow the pathological progression of latent AD and thereby prevent the onset of dementia [25]. However, the finding that indomethacin reduces cognitive decline in AD [26], has not been replicated by large-scale, randomized controlled trials. NSAIDs showed no clinical effects on cognitive outcomes in patients with prodromal symptoms or dementia of AD [4,27,28]. Furthermore, NSAID treatment for 1–3 years did not prevent or delay the onset of AD in elderly healthy individuals with a family history of AD, which was investigated by follow-up for 5–7 years after termination of the treatment, according to the Alzheimer’s Disease Anti-Inflammatory Prevention Trial (ADAPT) and follow-up study (ADAPT-FS) [4,29–31]. In contrast, Vlad et al. [32] prospectively assessed the effects on AD risk of NSAID use in subjects aged 50 years and older, free of AD at baseline, and found that long-term (>5 years) NSAID users had a lower risk of AD than non-NSAID users. In addition, retrospective studies have shown that treatment of rheumatoid arthritis with anti-inflammatory medication may reduce the risk of AD [26].

## Comparison of Cytokine Expression Levels between AD Patients and Non-demented Individuals with and without AD Pathology

### High pathology control (HPC)

It remains to be determined whether inflammatory alterations are the cause or consequence of neurodegeneration that leads to overt AD dementia, and whether anti-inflammatory therapy reduces cognitive impairment in AD patients in very early or preclinical stages. To address this issue, we compared the expression profiles of several cytokines in the temporal cortex of postmortem AD and control individuals [33]. There were two control groups: non-demented patients with increasing AD pathology, referred to as the HPC group, and normal individuals without dementia (ND) group. The ND group was also referred to as the low pathology control (LPC) group. LPC was defined clinically as being non-demented and pathologically as having the Consortium to Establish a Registry for AD (CERAD) neuritic plaque density scores [34] of none or sparse and Thal’s Aβ-phase [35] of 1 or 2, with Braak NFT stage [36] of I, II or III. LPC individuals may meet “low” probability of AD based on the National Institute on Aging (NIA)–Reagan Institute (RI) neuropathological criteria for AD [37], but exhibited no antemortem dementia symptoms. AD was defined clinically as being demented and pathologically as having CERAD neuritic plaque density scores of frequent and Thal’s Aβ-phase of 3, 4 or 5, with Braak NFT stage of V or VI. The AD patients are at the NIA–RI “high” probability of AD.

Our previous study [33] compared data from AD, HPC, and ND (LPC) individuals. The HPC group was defined clinically as being non-demented and pathologically as having CERAD neuritic plaque density scores of moderate or frequent and Thal’s Aβ-phase of 2 or 3, with Braak NFT stage of III or IV. Lue et al. [38,39] isolated HPC individuals that met “intermediate” probability of AD based on the NIA-RI pathological criteria for AD but exhibited no antemortem symptoms to otherwise qualify for the diagnosis of AD [40]. They found that HPC individuals had little or no neocortical or limbic synaptic loss and represented an intermediate subset between AD and LPC individuals [38,39]. To elucidate the role of inflammation in AD and promote the development of new treatments for early AD, data collected from HPC individuals are particularly important. HPC individuals are defined clinically and pathologically, while MCI is defined according to clinical criteria. The pathology of MCI is variable and not restricted to AD [40–42]. Some reports have described MCI as a clinical diagnosis, but the underlying neuropathological findings of amnestic MCI, in particular, are just being defined [43,44]. The temporal expression profiles of cytokines and other inflammatory molecules have been poorly investigated in AD postmortem brain tissues [45–47]. HPC cases have not been used much to study temporal expression profiles of inflammatory molecules involved in pathogenesis of AD, although many elderly people have AD pathological lesions but remain cognitively normal [48,49]. The temporal expression profiles of different kinds of Aβ and phosphorylated tau (p-tau) species have been demonstrated in postmortem brain samples of individuals with AD neuropathologic changes in the absence of cognitive impairment [38,50–53]. We believe that HPC cases represent a more accurate transition between normal aging and early stage AD to investigate whether neuroinflammation is a causes or consequence of neurodegeneration in AD.

### The objection to HPC

Some object to the use of HPC individuals as an intermediate subset between AD and ND (LPC) because HPC subset may imply a different pathogenesis from that of AD group. This is because HPC individuals meet the pathological criteria for AD but show no antemortem symptoms of dementia. However, HPC cases are not only intermediate between AD and LPC in terms of Aβ plaques or NFTs, but it is also intermediate in terms of the cortical cholinergic deficits [54–56]. In addition, HPC individuals have a significantly higher prevalence of the apolipoprotein (Apo) E ε4 allele [57] and toxic Aβ oligomers, although they do not bind to the postsynaptic site with the preservation of synaptic integrity [52]. These suggest that HPC would have progressed to AD with dementia if the patients had lived long enough.

### Cytokines attracting our attention in AD research

We have previously investigated the expression of the following cytokines [33]: (i) Interleukin (IL)-1β, IL-6 and tumor necrosis factor (TNF)α, which are frequently evaluated cytokines in AD [3,58,59]. (ii) Other members of the IL-1 superfamily, including IL-1 receptor antagonist, IL-18, and IL-33, which are involved in AD pathogenesis [17,60–65]. In addition, IL-1 is a master regulator of neuroinflammation [59,66]. (iii) Anti-inflammatory cytokines, IL-10 and IL-13, which had long been thought to participate in a counter-regulatory or reciprocal mechanism that protects the brain from detrimental proinflammatory mediators, but were recently shown to worsen AD-relevant phenotypes either dependently or independently of involvement of other inflammatory mediators [27,67–70]. (iv) IL-16, which is a lymphocyte chemoattractant factor and is correlated with AD [71,72]. (v) IL-32, a relatively novel cytokine that promotes the production of TNFα and is involved in AD pathogenesis [73]. (vi) IL-8 and monocyte chemotactic protein (MCP)-1, which are CXC and CC chemokines, respectively, and contribute to AD pathogenesis, shedding light on the development of a new strategy for treating AD with anti-inflammatory agents [4,74,75]. (vii) Macrophage migration inhibitory factor (MIF), a noncognate ligand of CXC chemokine receptors, which has attracted attention in AD research for the role in AD [76]. (viii) Converting enzymes for IL-1β, IL-18 and IL-33, and TNFα; that is, IL-1β-converting enzyme (ICE) and TNFα-converting enzyme (TACE), respectively. (ix) Transforming growth factor (TGF)β1, which is an important factor with beneficial and detrimental effects on AD pathogenesis [27,77,78].

### Evaluation of cytokine mRNA expression profiles in AD, HPC, and ND (LPC)

We have investigated the cytokine expression profiles in the brain using real-time PCR techniques [33]. Brain tissue samples were obtained from the Banner Sun Health Research Institute (BSHRI) Brain Donation Program [79]. In each sample, mRNA expression levels of each target cytokine were normalized to a reference housekeeping molecule, peptidyl-prolyl isomerase A (PPIA) (target cytokine to reference ratio; c/r ratio). In each patient, the normalized value (c/r ration) of cytokine expression in the temporal cortex that is vulnerable to AD pathology was divided by the c/r ratio in the cerebellum, where AD pathological changes are lowest [80,81]. The expression ratio of temporal cortex to cerebellum (T/Ce ratio) was compared among AD, HPC and ND individual groups. The demographics of these individuals are shown in Table S1. We found that the mRNA levels for IL-1β, IL-10, IL-13, IL-18, IL-33, TACE, and TGFβ1 were significantly higher in the temporal cortex of AD patients compared to HPC individuals. No cytokines had significantly lower mRNA levels in the temporal cortex of AD patients compared to HPC individuals. There were no significant differences in cytokine mRNA expression in the temporal cortex of HPC and ND individuals, or in the cerebellum of AD, HPC, and ND individual groups. TNFα mRNA expression was not significantly different in the temporal cortices of AD, HPC, and ND individuals, and was significantly higher in the cerebellum of AD compared to ND individuals [33].

### The protection of the cerebellum against AD pathology

The cerebellum generally lacks Aβ deposits in the AD brain, despite Aβ production in all brain regions [81]. Synaptic Aβ and p-tau, which finally form senile plaques and NFTs, are lowest in the cerebellum of AD cases [80]. In the cerebellum of AD patients used in our study, only sparse diffuse and cored plaques were observed (Table S1); that is, no typical plaques associated neuritic alterations, NFTs, or glial reactions were found, in agreement with previous findings [82–85]. Plaques were not observed in the cerebellum of HPC or ND individuals (Table S1). Therefore, inflammatory changes linked to AD pathology are very low in the cerebellum of AD patients [84]. However, the mechanisms that protect the cerebellum from AD pathology have not been clearly defined. Regional differences in immune-mediated reactions and pathological changes in the central nervous system (CNS) were proposed to play a role [83,84,86,87].

### The expression profiles of cytokine proteins

Our previous study [33] has demonstrated the following: (i) cytokine mRNA expression in the temporal cortex, which is vulnerable to AD pathology, is different between AD and HPC individuals but not between HPC and ND individuals; (ii) cytokine mRNA expression is not altered in the cerebellum of AD, HPC, and ND individuals, except TNFα. Cytokine expression in the HPC brain is more similar to ND than AD brains. Our finding that TNFα expression is different in the cerebellum of AD patients may provide the first immunological insights into the protection of the cerebellum from AD pathology. In our study, mRNA transcription levels were measured only in the brain. To confirm our findings, it is important to investigate changes in inflammatory mediators in postmortem brain tissues from AD, HPC, and ND individuals on the protein level. Wood et al. [5] reported differences in mRNA and protein expression of neurotoxic cytokines in the postmortem brain samples of AD patients and controls. We examined the expression of some inflammatory mediators at the protein level, including several proteins in the complement system, TNFα, and its receptors in the brain of AD patients compared to HPC and ND individuals as shown below.

To date, cytokine expression profiles have not been investigated at the protein level in HPC individuals [53]. A limited number of cytokine studies using postmortem brain tissues has focused on protein levels in AD patients compared to those in controls [46,47,88–90], although the findings were inconsistent [18,27]. Wood et al. [5] compared cytokine expression in different brain regions including the entorhinal cortex, amygdala, and superior frontal gyrus in postmortem AD and non-AD control brains. Instead of using HPC brains, they analyzed the expression in these three different brain regions, where AD lesions first appear at Braak NFT stage I–II, III–IV, and V–VI, respectively; therefore, these regions generally show relatively advanced, moderate, and mild AD pathology, respectively, at the time point when the postmortem brains are examined. Carriba et al. [88] demonstrated changes in TNFα signaling components in postmortem bran samples from individuals with no AD histopathological alterations (Braak 0), asymptomatic subjects with some AD histopatholical modification (Braak II), and demented AD patients (Braak V and VI). However, they did not include intermediate cases like HPC (Braak III and IV).

## Other Neuroinflammatory Mechanisms Involved in AD Pathogenesis

### Complement proteins

Many types of inflammatory mediators are involved in AD pathogenesis [3,4,8,18,58]. The production of complement proteins increases in AD [3]. Using immunohistochemistry, we demonstrated that C1q and the membrane attack complex (MAC) were present in Aβ plaques and tangled neurons in AD brains, whereas limited C1q and MAC immunoreactivity was observed in ND brains [91] (Table S2). In the HPC brain, minor MAC immunoreactivity was observed; however, C1q immunoreactivity was clearly observed in a fraction of Aβ plaques and tangled neurons [91] (Table S2). These findings were confirmed using Western blotting by Lue et al. [39]. Thus, these data suggest that the complement system is noticeably involved in later stages of AD but may be initially activated at earlier stages of the disease.

### CD59

CD59 is expressed in the brain [92,93]. Decreases in CD59 expression promote MAC formation [94,95]. MAC expression is upregulated in the AD brain [91]. We observed CD59 expression in many neurons in ND brains, while expression almost disappeared in AD brains. In the HPC brain, neurons were partly positive for CD59 (manuscript, in preparation). This indicates an earlier involvement of CD59 in the CNS damage and pathology caused by AD. In contrast, Yasojima et al. [96] previously demonstrated positive bands for CD59 on Western blots found in both AD and normal hippocampal tissues.

### Stress hormones

The elevation of glucocorticoids (GCs) secreted by the adrenal glands upon chronically stressful stimuli that occurs during aging may accelerate the progression of brain aging, particularly the hippocampus. Although under physiological stress GCs generally exert anti-inflammatory effects in the CNS, the chronically elevated GC levels under stressful stimuli can potentiate neuroinflammation and increase the expression of proinflammatory cytokines, such as IL-1β and TNFα, which in turn activate many signaling pathways, including nuclear factor kappa-B (NF-κB), and microglia function [97,98]. As a result, GCs increase the vulnerability of neurons to numerous chronic stressful events, including metabolic, ischemic, excitotoxic, oxidative, and infectious insults. Thus, the aging process is facilitated in stress-vulnerable neurons in the cerebral cortex and hippocampus, which leads to neuronal death [98–101]. Neuroinflammation is triggered by chronic stress in a GC receptor (GR)-dependent manner, and the reduced expression of hippocampal GRs is associated with an exaggerated response to chronic stress. These data confirm the negative feedback inhibition of hippocampal projections on the hypothalamic-pituitary-adrenocortical axis and the consequent elevation and damaging effects of circulating GCs [97,102].

Hippocampal changes arising from chronic stress via the long-term elevation of GC signaling are also evident in AD-associated processes, including aging [8,97]. Indeed, aging human subjects exhibit higher mean diurnal values of GCs, and some subjects with higher GCs later develop AD. Chronic GC administration to aged monkeys and transgenic mouse models of AD increased Aβ pathology and led to memory impairment. These data suggest that excess GCs drive important components of the declining brain function observed in AD and during the aging process [98]. GCs are hormones that are released during the stress response and are well known for their immunosuppressive and anti-inflammatory properties [102]; however, recent studies have uncovered examples of contrasting effects, as observed in adrenalectomized/GC-supplemented rats [103] and a randomized controlled trial of low-dose predonisone in AD patients [104].

## Anti-inflammatory Therapy for AD (1)

### Non-steroidal anti-inflammatory drugs (NSAIDs)

Aisen et al. [104] suggested that with more limited effects of NSAIDs on inflammation than those of GCs, and without the issue of possible hippocampal toxicity, NSAIDs would be appropriate candidates for clinical trials in AD. Substantial evidence from laboratory and epidemiologic studies has suggested that anti-inflammatory medication, especially NSAIDs, can defer or prevent AD occurrence. However, recent meta-analyses concluded that ADAPT and ADAPT-FS [29,105] did not support the use of NSAIDs for AD prevention among dementia-free individuals. The ADAPT study was designed to evaluate the efficacy of naproxen, a non-selective cyclooxygenase (COX) inhibitor, and celecoxib, a selective COX-2 inhibitor, in treating AD [105]. Many reasons have been proposed for the discrepancies between the findings of observational studies and ADAPT. These include the choice of specific NSAIDs, duration of treatment, ApoE ε4 allele, age, disease stage, and speed of disease progression [27,29,105,106].

NSAIDs reduce neuroinflammation, Aβ levels, AD-like brain pathology, and behavioral impairments in mouse models of AD via several pathways [107–110]. Because upregulated COX-2 is implicated in AD pathogenesis, NSAIDs exert anti-inflammatory effects possibly by blocking COX-2 in the brain [105,111]. NSAIDs may affect Aβ production by suppressing microglial activation or modifying γ-secretase activity, thereby reducing Aβ42 levels. Furthermore, NSAIDs may reduce β-secretase levels by activating the nuclear receptor peroxisome proliferator-activated receptor γ (PPARγ), thereby decreasing Aβ levels [112].

According to Weggen et al. [113], the non-selective COX inhibitors ibuprofen and indomethacin reduced Aβ42 secretion from cultured cells. However, naproxen, which belongs to the same class of drugs as ibuprofen, did not lower Aβ42 levels in culture medium [113] and was associated with increased Aβ pathology [114]. They also reported that SC-560 (a selective COX-1 inhibitor) and celecoxib did not lower Aβ42 secretion, concluding that the capacity of a subset of NSAIDs, including ibuprofen and indomethacin, to reduce Aβ42 levels was independent of COX activity [110,113,115]. COX-2 expressed in neurons may have a neuroprotective function; therefore, COX-2 inhibition may have deleterious consequences in AD treatment trials [58,106,116]. Several COX-2 inhibitors, including celecoxib, were reported to increase Aβ42 levels [115,116]. The selective COX-2 inhibitor rofecoxib possibly accelerated underlying AD neuropathology in individuals with MCI [117]. There was a contrasting outcome where celecoxib use possibly improved cognitive performance in people with relatively mild age-related cognitive complaints, before subclinical neuropathological changes emerged [118]. Conversely, COX-1 highly expressed in microglia has been suggested to be a more appropriate target for anti-inflammatory therapies for AD [58,106,119], as it is associated with a significant reduction in the probability of progression from MCI to AD [120].

Kotilinek et al. [121] proposed a third possible mechanism by which NSAIDs may protect against AD, involving the blockade of a COX-2-mediated prostaglandin E2 (PGE2) response at synapses. This block occurred independently of reductions in Aβ42 or decreases in inflammation.

### Inflammation and defective insulin signaling in AD

AD is a multifactorial neurodegenerative disease, and inflammation is an integral part of its pathogenesis [3,8]. A host of comorbid conditions are associated with aging, including metabolic endotoxemia, type 2 diabetes (T2DM), prediabetic hyperglycemia, obesity, dyslipidemia, metabolic syndrome, and chronic disorders of the respiratory and cardiovascular systems. All of these conditions upregulate systemic inflammation [8,122–125]. Inflammation is a basic part of the body’s defense mechanisms against multiple threats [125]; however, the prolonged dysregulation of this system, which can occur during aging, induces the production of neurotoxic proinflammatory cytokines and GCs that promote neuroinflammation and exacerbate neurodegeneration, which ultimately leads to cognitive decline and AD [8]. Compared with younger individuals, older healthy individuals are more likely to suffer profound memory impairments following the neuroinflammatory response to challenging life events [124,126]. These age-related conditions are linked to insulin resistance, which is a condition with chronic low-grade inflammation as an essential pathogenic factor [8,127]. Proinflammatory cytokines can cause insulin resistance [7]. Numerous epidemiological, clinical, and experimental studies have strengthened the link between T2DM and AD. Several pathological features, including insulin resistance, disrupted insulin signaling, and inflammation, appear to be shared by patients with T2DM and AD [123,125]. Bomfim et al. [128] demonstrated that mechanisms analogous to those that account for peripheral insulin resistance in patients with T2DM underlay the impaired insulin signaling and neuronal dysfunction in the brains of AD patients. Aβ oligomers (AβOs), synaptotoxins that accumulate in AD brains, impaired neuronal insulin signaling via activation of TNFα and stress-related kinases, including the IκB kinase (IKK), c-Jun N-terminal kinase (JNK), and double-stranded RNA-dependent protein kinase (PKR), which induced the phosphorylation of eukaryotic translation initiation factor 2α (eIF2α) and caused serine phosphorylation of insulin receptor substrate (IRS)-1 (IRS-1pSer), while inhibiting the physiological tyrosine phosphorylation of IRS-1 (IRS-1pTyr). IRS-1pSer, but not IRS-1pTyr, interfered with the ability of IRS-1 to engage in insulin receptor (IR) signaling, thus blocking the intracellular action of insulin [128]. The phosphorylation of IRS-1 at tyrosine residues is required for insulin-stimulated responses, whereas the phosphorylation of IRS-1 at multiple serine residues causes a reduced insulin response that is consistent with insulin resistance [129]. Insulin signaling is generally involved in brain glucose uptake and utilization, energy production, neuronal survival, synaptic plasticity, gene expression, and learning and memory [130–132]. Interestingly, the induction of IRS-1pSer by AβOs in neurons was blocked by infliximab, a TNFα neutralizing antibody, and exendin-4 (exenatide), a novel anti-diabetic drug that activates insulin signaling through stimulation of glucagon-like peptide 1 (GLP-1) receptors. The reduction of IRS-1pSer reduced amyloid pathology, decreased synapse loss, and improved cognition in transgenic mouse models of AD [128,133]. These results were confirmed by PKR and TNF receptor (TNFR) knockout mice [134]. The enzyme dipeptidyl peptidase-4 (DPP-4) degrades endogenous GLP-1, and DPP-4 inhibitors attenuated Aβ-induced cytotoxicity *in vitro* [7,135]. These results support research into the repurposing of diabetes drugs for brain insulin resistance in AD [7,124,129]. Notably, insulin prevented the increase in IRS-1pSer induced by AβOs [128], and intranasally administered insulin improved cognition in individuals with MCI and early-stage AD [7,136].

Talbot et al. [137] demonstrated that the AD brain exhibited reduced response to insulin signaling in the IR/IRS-1/ phosphatidylinositol 3-kinase (PI3K)/Akt pathway. This reduced insulin response may lead to increased glycogen synthase kinase-3 (GSK-3) activity, which is involved in the hyperphosphorylation of tau, Aβ production, neuroinflammation, glial activation, apoptotic neuronal death, and memory impairment in AD [7,129,138,139].

## Involvement of TNFα in AD Pathogenesis

### The mechanisms of its dual function

TNFα is centrally involved in the pathogenesis of AD [140–142] and is one of the most investigated cytokines in the CNS [18]. Astrocytes and microglia are classically believed to serve as the predominant sources of TNFα in the CNS. Furthermore, neurons express this cytokine in pathological states in the CNS [143].

TNFα signals exert bidirectional effects of being supportive and harmful to neuronal function [3]. TNFα is associated with Aβ plaque formation [142]. Aβ in plaques stimulates microglial and astrocytic secretion of TNFα [144,145]. It remains unclear whether microglial and astrocytic responses are protective or deleterious [5,58]. Moreover, TNFα is implicated in neuronal death in AD [88,146]. TNFα signaling enhances Aβ production and Aβ- and glutamate-induced neurotoxicity [134,147–149]. Wood et al. [5] revealed that TNFα was a potent inducer of neuronal death and reduced neuronal viability *in vitro*, when applied either alone or together with Aβ. Janelsins et al. [143] demonstrated that TNFα expression for 4 months specifically in hippocampal neurons of triple-transgenic AD (3xTg-AD) mice by a recombinant adeno-associated virus (rAAV) expressing human TNFα enhanced intracellular levels of Aβ as well as microglial activation. More prolonged expression for 10 months induced extensive microglial activation, pro-apoptotic signaling cascades, and neuronal death, accompanied with reduction in extracellular Aβ plaque load. This indicates that chronic, neuron-specific TNFα expression induced neuronal death where the coincident expression of the pathogenic transgene products of 3xTg-AD mouse neurons was required. Several anti-TNFα biologic medications have attenuated Aβ deposition, behavioral impairments, and inflammation in AD animal models [27]. Inhibiting TNFα signaling reduced Aβ generation and plaque formation, and it prevented learning and memory deficits in APP23 transgenic mice [150]. Inhibition of soluble TNF signaling prevented pre-plaque AD pathology in 3xTg-AD mice exposed to chronic systemic inflammation [151].

On the other hand, Chakrabarty et al. [152] reported that hippocampal expression of TNFα by transduction of rAAV expressing mouse TNFα in APP transgenic TgCRND8 mice induced glial activation, attenuating Aβ plaque formation without causing bystander neurotoxicity. More lines of studies have demonstrated that TNFα protected neurons from Aβ toxicity [153–155] and metabolic-excitotoxic insults [156,157]. The long form of Fas apoptotic inhibitory molecule (FAIM-L) is a Fas antagonist and is expressed exclusively in neurons. FAIM-L expression promoted TNFα-mediated protection against Aβ-induced neuronal death [88].

These findings show that excess TNFα signaling has detrimental and beneficial effects on neurons. This depends on many factors, including the presence or absence of Aβ [143]; aging [158]; the type of TNFR predominantly expressed and activated to trigger downstream signaling pathways [3,159]; downstream intracellular signaling molecules such as NF-κB, CREB-binding protein (CBP), FAIM, and cellular inhibitor of apoptotic protein (cIAP), which are predominantly recruited when TNFα binds to its receptors [88,153,155,160,161]; the balance of soluble and transmembrane forms of TNFα [162]; the presence of soluble TNFRs (sTNFRs) to neutralize or decrease TNFα signal [159–161]; cell type and activation state; intra- and extra-cellular environments; inflammatory milieu [163,164]; and crosstalk between glutamate receptors and their signaling pathways [148,165,166].

### TNFα levels in AD

Several cytokines including TNFα are expressed in a disease progression-dependent manner; that is, they increased steadily or peaked when MCI progressed to AD, and therefore, may represent suitable molecules for disease prediction [27]. In a literature review, Brosseron et al. [18] analyzed the expression of 66 cytokines in the serum, plasma and CSF of AD and MCI individuals compared to normal control subjects. They reported inconsistent TNFα levels in serum, plasma, and CSF of AD and MCI individuals. TNFα expression was upregulated, downregulated, or not regulated in the reviewed studies. In the recent meta-analysis, no significant differences in TNFα levels were found between MCI and healthy control subjects [167]. TNFα expression in the AD brain generally seems to be upregulated [5,142,147], although some reports have demonstrated its decline in AD [46,168].

### TNFα-converting enzyme (TACE)

TNFα is produced as a membrane-bound precursor molecule (mTNFα) of 26 kDa, which is cleaved by TACE to produce a soluble 17 kDa mature cytokine (sTNFα) [142]. Both forms of TNFα are active and have distinct biological activities [169]. It was reported that TACE is the α-secretase responsible for the majority of regulated α-cleavage of APP, suggesting that alterations in TACE activity during aging may contribute to Aβ formation [170]. The two types of TNFRs are also thought to be substrates of TACE, which cleaves TNFRs, releasing sTNFRs that regulate TNFα signaling by shedding of cell surface TNFRs [171]. Jiang et al. [172] reported elevated TACE and sTNFRs levels in the CSF of MCI and AD patients. Kim et al. [173] showed that TACE inhibition mitigated the effects of TNFα in the AD brain.

### Our data on TNFα protein levels in AD, HPC and ND (LPC)

In this section, we present our data on TNFα expression normalized to glyceraldehyde-3-phosphate dehydrogenase (GAPDH) and β-actin, in the AD brain. These were compared to levels in the brain of HPC and ND individuals. Expression was measured in the temporal cortices and cerebella of autopsied brain samples from five AD patients, and five age and gender-matched HPC and ND individuals each. The demographic characteristics were similar to those shown in Table S1. Brain samples were provided by the BSHRI brain bank. The postmortem intervals (PMIs) averaged less than 4 hrs.

**Figure 1:**
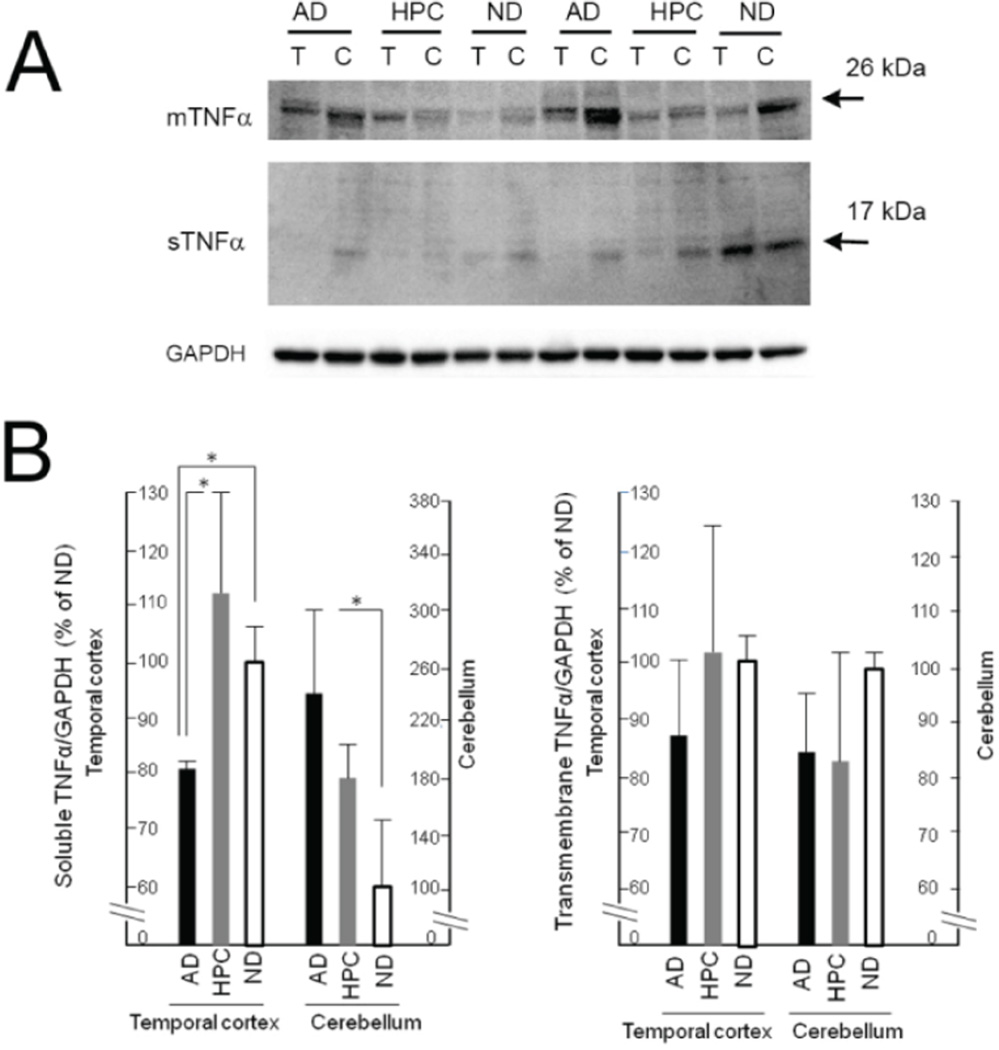
TNFα protein levels in the temporal cortex and cerebellum of the AD, HPC and ND Brain. **(A)** Representative images of soluble TNFα (sTNFα) and transmembrane TNFα (mTNFα) protein bands at approximately 26 kDa and 17 kDa, respectively, from Western blots. T: Temporal cortex; C: Cerebellum; GAPDH: Glyceraldehyde-3-phosphate dehydrogenase. **(B)** Densitometry analysis of sTNFα and mTNFα protein expression in the AD, HPC and ND brain. Data on the protein levels of sTNFα and mTNFα were normalized to GAPDH. Subsequently, the normalized data of AD and HPC groups were compared with ND group, and expressed as relative expression levels, where the data on ND were set as 100%. Values are expressed as the mean ± S.D. of the relative expression levels. n=5, *p<0.05 by Kruskal-Wallis non-parametric analysis with Steel-Dwass post-testing.

Mature sTNFα and precursor mTNFα were recognized with the anti-TNFα antibody (R&D Systems, AF210NA) as positive bands at approximately 17 kDa and 26 kDa [174], respectively, on Western blots (Figure 1). The expression of sTNFα differed significantly in the cerebellum as well as in the temporal cortex of AD, HPC, and ND individuals. Differences were observed between the AD and ND groups (AD<ND) and the AD and HPC groups (AD<HPC) in the temporal cortex, and between the AD and ND groups (AD>ND) in the cerebellum (p<0.05; Kruskal–Wallis non-parametric analysis with Steel–Dwass post-testing). No significant differences in sTNFα levels were observed between the HPC and ND groups. There were no significant differences in mTNFα levels across the three individual groups (Figure 1).

Our data indicate that TNFα protein expression was lower, not higher, in the vulnerable temporal cortex of AD patients compared to HPC or ND individuals. Previous findings regarding TNFα expression in the AD brain have been inconsistent. Lanzrein et al. [46] reported its decrease in AD. Differences have also been reported between gene and protein expression [5]. The discrepancy between TNFα mRNA [33] and protein expression, particularly in the vulnerable temporal cortex, needs further investigation in more postmortem brain samples. The different levels of sTNFα and mTNFα proteins may be explained by different levels of TACE. TACE levels were reported to increase in AD and MCI individuals and were significantly higher in MCI than in AD [172]. We observed that TACE mRNA expression increased in AD [33].

## TNF Receptors (TNFRs) in AD Pathogenesis

### Each TNFR has a dual function

The biological effects of TNFα are exerted by its binding to TNFR type 1 (TNFR1) and TNFR type 2 (TNFR2) [159]. TNFR1 is responsible for the majority of biologic actions of sTNFα [174]. TNFR2 can only be fully activated by mTNFα, but not sTNFα [161].

The main receptor for TNFα, TNFR1 can trigger two signaling pathways, leading to survival and death. Survival signaling begins with complex I formation, consisting of TNFR1, TNFR-associated death domain protein (TRADD), receptor-interacting protein 1 (RIP1) and TNFR-associated factor 2 (TRAF2), followed by the activation of NF-κB, which leads to the transcriptional activation of pro-survival genes, such as those encoding cIAP1/2, Bcl-2, Bcl-xL, and FLICE-like inhibitory protein (FLIP). Death signaling is initiated by recruitment of TRADD and RIP1 to TNFR1, which bind to the Fas-associated death domain protein (FADD) after the dissociation from TNFR1. This recruits and activates caspase-8 to finally form complex II in instances where the complex I or NF-κB fails to be activated [88,175,176]. Del Villaer and Miller [177] demonstrated that differentially expressed in normal versus neoplastic (DENN)/MAPK activating death domain (MADD) protein, a TNFR1 binding protein, was down-regulated by Aβ in hippocampal neurons in AD patients and APP transgenic mice, allowing the TRADD-mediated TNFR1 apoptosis signaling. Carriba et al. [88] reported that Aβ reduced the expression of FAIM-L exclusively localized in neurons, thereby shifting the TNFα-mediated inflammatory response from neuronal protection to death.

In general, TNFR2 is believed to mediate its effects by intensive crosstalk with TNFR1 signaling pathways and ligand passing to neighboring TNFR1 [159,160,162]. However, these are not the only function of TNFR2. TNFα can signal through TNFR2 that lacks a death domain, recruiting TRAF2, TRAF1, and cIAP1/2, and activating the JNK/cJun pathway, which stimulates pro-apoptosis gene expression, although JNK can also inhibit induction of apoptosis by TNFα [178]. Signaling through the TNFR2 pathway is thought to play protective roles in several disorders, including neurodegenerative disorders. In contrast, several mechanisms can account for TNFR2-mediated apoptosis [160].

Signaling pathways initiated by TNFRs may either cooperate or counterbalance each other [159]. TNFα binds to TNFR 1 and TNFR2, generally relying on TNFR2 for survival and on TNFR1 for apoptosis. However, research has shown some degree of overlap in the function of the two receptors. It is not always true that TNFR1 functions for apoptosis while TNFR2 for survival. As described above, many factors can affect TNFR1 versus TNFR2 signaling [160].

Of interest, TNFRs can have counteracting functions, at least in neural tissues [161]. Fontaine et al. [179] demonstrated that, in a mouse model of retinal ischemia, TNFR1 contributed to cell death, whereas TNFR2 promoted cell protection dependent on Akt/protein kinase B (PKB) pathway. Marchetti et al. [166] demonstrated that TNFR2-mediated neuroprotection against glutamate-induced excitotoxicity was exerted by persistent activation of NF-κB in a PI3K-dependent manner. This was counterbalanced by the action of TNFR1, which only induced rapid but transient NF-κB activation, leading to cell death. These results strongly suggest that anti-TNF therapies could be made successful if they selectively target TNFR1 signaling by efficient neutralization of sTNFα without eliciting collateral damage to TNFR2/mTNFα-dependent processes [162].

### The role of TNFRs in mouse models of AD

Mice that globally lack both TNFRs showed exacerbation of excitotoxic and ischemic neuronal injuries accompanied by a reduced response of microglia to excitotoxins and ischemia, indicating a neuroprotective role of TNFα signaling [180]. Long-term, global ablation of both TNFRs in all cell types in 3xTg-AD mice exhibited enhanced amyloid and tau-related AD pathology and reduced Aβ phagocytic activity of microglia, arguing against the long-term use of pan-anti-TNFα inhibitors for treating AD [181].

Detrimental and neuroprotective roles have been reported for TNFα signaling, illustrating the complexity of these signaling pathways [163]. TNFR1 deletion in APP23 transgenic mice reduced Aβ generation, lessened neuronal loss, and alleviated Aβ-related learning and memory deficits [150]. Deletion of TNFR2 in APP23 transgenic mice increased Aβ production, plaque formation, and microglial activation [182]. These two reports suggest that the two TNFRs have opposite contributions to AD pathology.

Montgomery et al. [163] demonstrated that rAAV2 vector-mediated neuron-selective knockdown of TNFR2, but not TNFR1 or TNFR1 + 2, in 3xTg-AD mice led to a significantly dramatic enhancement of Aβ42 plaque deposition in a stage-dependent manner. This suggests the following: (i) the signaling dichotomy between TNFR1 and TNFR2 also exists in disease; (ii) unopposed neuron-specific TNFR1 signaling at later stages of disease, when TNFR2 has been selectively down-regulated, increases the severity of AD pathogenesis; and (iii) TNFR2 exerts protective action that may be required to counteract TNFR1 signaling, emphasizing the need for strategies that more selectively modulate TNFα signaling in specific cell types and at different stages of disease.

### Our data on TNF receptor levels in AD, HPC and ND (LPC)

We demonstrated that expression of cytokines including sTNFα in the HPC brain was closer to ND than AD brains. We next compared TNFR1 and TNFR2 expression in the brains of AD patients and compared this to HPC and ND brains. This study used postmortem tissue samples from the vulnerable temporal cortex and resistant cerebellum of ten AD patients and ten age and gender-matched HPC and ND individuals each. Demographic characteristics were similar to those shown in Table S1. The brain samples were provided by the BSHRI brain bank. PMIs averaged less than 4 hrs. The anti-TNFR1 (Gene Tex, GTX27630) and anti-TNFR2 (Cell Signaling, 3727) antibodies were used for Western blot analyses. TNFR protein levels in vulnerable brain areas, including the frontal and temporal cortices and the hippocampus of AD and ND individuals have already been reported using autopsied human tissues [183,184]. However, they did not use brain samples of HPC subjects. It is important to examine TNFR expression in the HPC brain to determine whether TNFR-mediated signaling primarily contributes to AD pathology prior to the manifestation of overt dementia, or is only a secondary response to AD pathology at later stages. To normalize the expression levels of target proteins, we used GAPDH and β-actin. In addition, the data normalized with GAPDH and β-actin were compared to data normalized with two neuronal cell body markers, neuron specific enolase and class III β-tubulin, as TNFR1 and TNFR2 are mainly expressed in neurons in the CNS [183,184]. All normalized data showed the same trend, unless otherwise stated.

**Figure 2:**
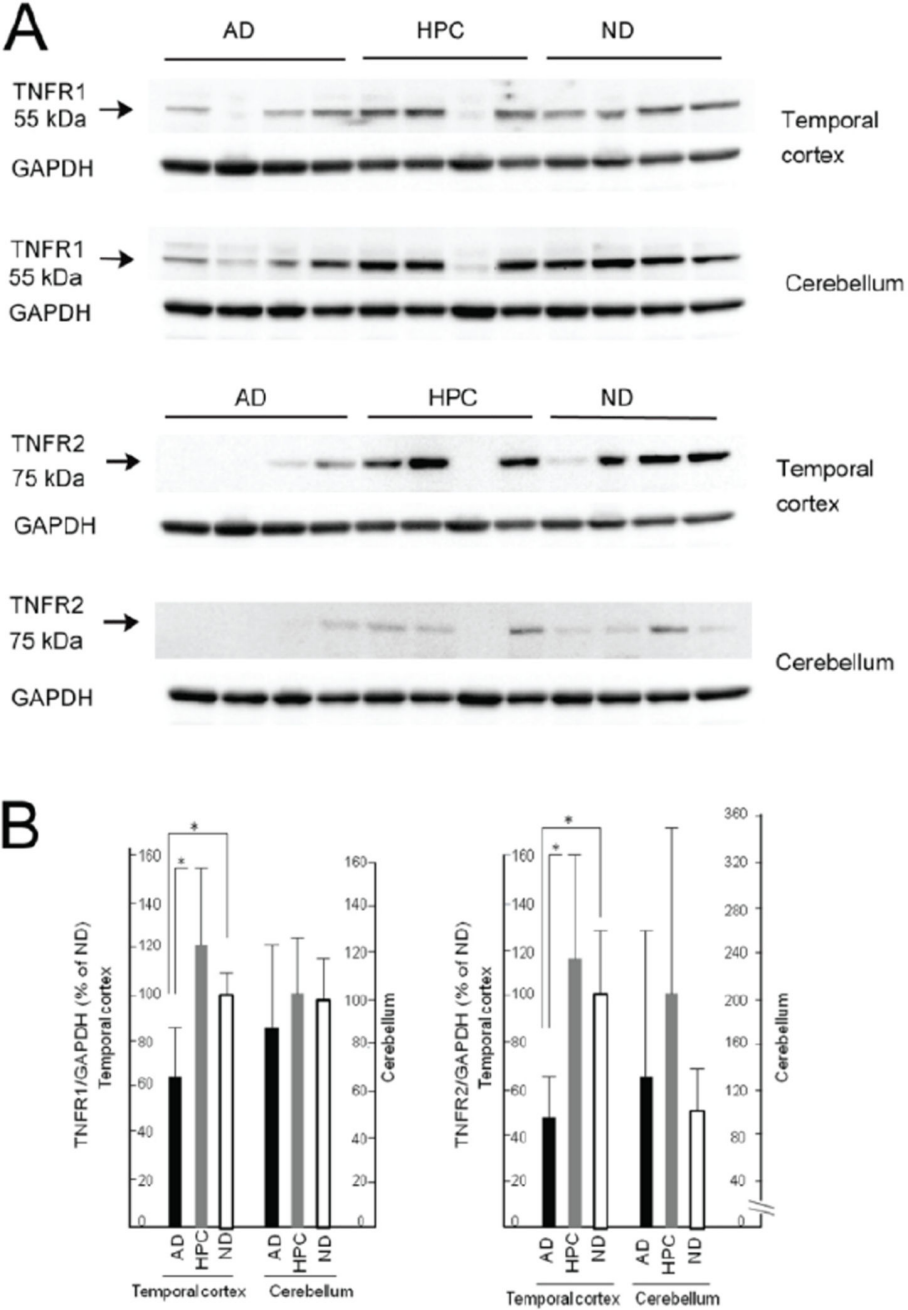
Protein levels of TNFR1 and TNFR2 in the temporal cortex and cerebellum of the AD, HPC and ND brain. **(A)** Representative images of TNFR1 and TNFR2 protein bands at approximately 55 kDa and 75 kDa, respectively, from Western blots. GAPDH: glyceraldehyde-3-phosphate dehydrogenase. **(B)** Densitometry analysis of the two TNFR proteins in the temporal cortex and cerebellum of the AD, HPC and ND brain. Data on the protein levels of TNFR1 and TNFR2 were normalized to GAPDH. Subsequently, the normalized data of AD and HPC groups were compared with ND group, and expressed as relative expression levels, where the data on ND were set as 100%. Values are expressed as the mean ± S.D. of the relative expression levels. n=10, *p<0.05 by Kruskal-Wallis non-parametric analysis with Steel-Dwass post-testing.

TNFR1 and TNFR2 were detected as single bands at approximately 55 kDa and 75 kDa, respectively (Figure 2). In the temporal cortex, densitometric analyses revealed that the expression levels of TNFR1 and TNFR2 were significantly different between the AD and HPC groups (AD<HPC) and the AD and ND groups (AD<ND) (p<0.05; Kruskal–Wallis non-parametric analysis with Steel–Dwass post-testing). No significant differences in the expression levels of both TNFRs were observed between the HPC and ND groups. In the cerebellum, there were no significant differences across the three groups. Our data indicate the following: (i) the expression levels of both TNFRs in the HPC group were closer to those in ND group than AD group in the temporal cortex; (ii) the expression levels of both TNFRs were lowest in the AD group in the temporal cortex; and (iii) both TNFR’s expression did not differ across the three groups in the cerebellum.

Our study is the first to investigate TNFR protein expression in HPC cases. We demonstrated lower levels of both TNFRs in AD, but not HPC individuals, compared to ND individuals. This means that alterations in immunological conditions involving TNFR signaling are not the primary events initiating AD pathological changes such as Aβ plaque and NFT formation but may be either early events occurring along with synaptic and neuronal changes or later events occurring as a secondary response to synaptic loss and neuronal death. As shown in Figure 2, TNFR protein levels may have tended to be elevated in HPC individuals compared to individuals with AD and ND, suggesting that TNFR signaling may play a role in the HPC stage, although this was not statistically significant. To validate our data, temporal expression patterns in ND, HPC, early stage of AD (Braak III–IV), and late stage of AD (Braak V–VI) need to be investigated. This was partly conducted by Carriba et al. [88] and González et al. [185], but these studies did not investigate HPC brains. Culpan et al. [184] demonstrated in their Figure 2 that levels of TNFR1 were lower in AD brains than control brains, and the differences were greater in the frontal cortex than the temporal cortex and hippocampus. In the study by Cheng et al. [183], TNFR1 protein levels were higher in the frontal tissues of AD brains compared to ND brains, and TNFR2 protein levels were lower in the frontal tissues of AD brains compared to ND brains. The discrepancy between our data and the data of Cheng et al. [183] may partly be explained by the fact that their AD cases were chosen randomly from the BSHRI brain bank and therefore were likely to be a mixture of intermediate (Thal’s Aβ-phase 4 or less, Braak NFT stage III–IV) and high (Thal’s Aβ-phase 5, Braak NFT stage V–VI) AD pathology, while our AD cases were all high and our HPC cases were all intermediate based on the NIA–RI criteria for AD [37]. TNFR2 protein expression was not different in cerebellum of AD brains compared to ND brains in our study. Therefore, the cerebellum may not be protected from AD pathology solely by TNF-α binding to TNFR2 as a survival signal.

## Anti-inflammatory Therapy for AD (2)

### TNFα as a potential target for AD therapy

There are many therapeutic targets in the neuroinflammation-related pathological processes leading to AD. These include cytokines, the complement system, GCs, IR signaling, glial activation, and other inflammatory mediators. Therefore, a multicomponent treatment approach (polypharmacy) may be required [8]. In each component of polypharmacy, strategies need to be developed to more selectively modulate immune signaling in specific cell types and at different disease stages [27].

TNFα is associated with pathological mechanisms that participate in neurotoxicity and neuronal damage in AD, implicating anti-TNF-α agents in AD therapy [4,5,8,27,141,147,160,162]. However, it is unclear whether TNFα signaling is toxic or trophic in AD pathological processes. Nonetheless, we expect anti-TNFα agents to be effective against AD [141,147]. Furthermore, given the restricted cellular distribution of TNFR2 in contrast with the widespread expression of TNFR1, the possibility of specifically targeting TNFR2 has emerged as a safer and more efficient therapeutic approach [160], although anti-TNFα therapies selectively targeting TNFR1 signaling have also been considered [162].

Anti-TNFα agents, including infliximab and etanercept, have been developed to specifically inhibit TNFα [141,147,162]. Intracerebroventricular infusion of infliximab, a murine-human chimeric bivalent anti-TNFα monoclonal antibody, in AD transgenic mice reduced the number of amyloid plaques [125]. Cognitive improvement was also reported in a woman with AD after intrathecal administration of infliximab [186].

Etanercept is a recombinant dimeric fusion protein comprising the extracellular ligand-binding portions of soluble human TNFR2 linked to the Fc fragment of human IgG1. It binds to mTNFα and trimeric sTNFα, and inhibits signaling through both TNFRs [27,162,187]. In 2006, a six-month, prospective, open-label, single-center, phase II pilot study investigated the effectiveness of perispinal etanercept injections in 15 patients with mild-to-severe AD. The author reported a significantly rapid and sustained cognitive improvement [147], which has been confirmed by other studies [188,189]. Furthermore, some AD patients treated perispinally with etanercept for more than five years exhibited sustained clinical improvement [187]. However, the effects of perispinal etanercept have not been supported by randomized, double-blinded, placebo-controlled studies on AD patients who were given peripherally (subcutaneously)-delivered etanercept [147,190]. Neither etanercept nor infliximab crosses the blood–brain barrier, thus requiring invasive forms of central administration [125].

Non-specific inhibitors of TNFα, such as thalidomide and minocycline, have been tested in AD treatment [7,191–193].

Blocking all effects of TNFα by anti-TNFα treatment can be counter-productive. A more effective therapeutic approach is the selective blocking of TNFα signaling; therefore, specifically targeting TNFR1 or TNFR2 as a new therapeutic approach is now being considered [160,194,195].

## Conclusions

Increasing evidence suggests that inflammatory mediators, including TNFα and its receptors, are involved in the pathogenesis of AD. However, findings regarding their function and expression in AD have been inconsistent. NSAIDs may provoke the contrasting effects of neuroinflammatory molecules at different stages of AD pathogenesis. Different AD stages may have different detrimental and protective factors. These illustrate the complexity of signaling pathways in the CNS, where neuroinflammatory signaling leads to either protective or detrimental outcomes, depending on a variety of conditions. To better define signaling mechanisms and therapeutic strategies for AD, temporal expression profiles of signaling molecules need to be elucidated in ND (LPC), HPC, early stage AD, and late stage AD brains. Expression also needs to be compared between vulnerable and resistant brain areas.

Very recently selective amplification of regulatory T cells by low-dose IL-2 treatment was shown to increase the number of plaque-associated microglia and restore cognitive function in APP/PS transgenic mice [196]. To date, the role of IL-2 in AD has poorly been discussed [1–5].

## References

[1] Weeraratna AT, Kalehua A, Deleon I, Bertak D, Maher G (2007). Alterations in immunological and neurological gene expression patterns in Alzheimer’s disease tissues. Exp Cell Res.

[2] Eikelenboom P, Bate C, Van Gool WA, Hoozemans JJ, Rozemuller JM (2002). Neuroinflammation in Alzheimer’s disease and prion disease. Glia.

[3] Akiyama H, Barger S, Barnum S, Bradt B, Bauer J (2000). Inflammation and Alzheimer’s disease. Neurobiol Aging.

[4] Heneka MT, Carson MJ, El Khoury J, Landreth GE, Brosseron F (2015). Neuroinflammation in Alzheimer’s disease. Lancet Neurol.

[5] Wood LB, Winslow AR, Proctor EA, McGuone D, Mordes DA (2015). Identification of neurotoxic cytokines by profiling Alzheimer’s disease tissues and neuron culture viability screening. Sci Rep.

[6] Czirr E, Wyss-Coray T (2012). The immunology of neurodegeneration. J Clin Invest.

[7] Clark I, Atwood C, Bowen R, Paz-Filho G, Vissel B (2012). Tumor necrosis factor-induced cerebral insulin resistance in Alzheimer’s disease links numerous treatment rationales. Pharmacol Rev.

[8] Daulatzai MA (2016). Fundamental role of pan-inflammation and oxidative-nitrosative pathways in neuropathogenesis of Alzheimer’s disease. Am J Neurodegener Dis.

[9] Abbayya K, Puthanakar NY, Naduwinmani S, Chidambar YS (2015). Association between Periodontitis and Alzheimer’s Disease. N Am J Med Sci.

[10] Sparks Stein P, Steffen MJ, Smith C, Jicha G, Ebersole JL (2012). Serum antibodies to periodontal pathogens are a risk factor for Alzheimer’s disease. Alzheimer’s Dement.

[11] McNaull BB, Todd S, McGuinness B, Passmore AP (2010). Inflammation and anti-inflammatory strategies for Alzheimer’s disease--a mini-review. Gerontology.

[12] Maccioni RB, Rojo LE, Fernández TA, Kuljis RO (2009). The role of neuroimmunomodulation in Alzheimer’s disease. Ann N Y Acad Sci.

[13] Huang Y, Mucke L (2012). Alzheimer mechanisms and therapeutic strategies. Cell.

[14] Weissmiller AM, Wu C (2012). Current advances in using neurotrophic factors to treat neurodegenerative disorders. Transl Neurodegener.

[15] Hardy J (2009). The amyloid hypothesis for Alzheimer’s disease: a critical reappraisal. J Neurochem.

[16] Rodriguez-Vieitez E, Saint-Aubert L, Carter SF, Almkvist O, Farid K (2016). Diverging longitudinal changes in astrocytosis and amyloid PET in autosomal dominant Alzheimer’s disease. Brain.

[17] Saresella M, La Rosa F, Piancone F, Zoppis M, Marventano I (2016). The NLRP3 and NLRP1 inflammasomes are activated in Alzheimer’s disease. Mol Neurodegener.

[18] Brosseron F, Krauthausen M, Kummer M, Heneka MT (2014). Body fluid cytokine levels in mild cognitive impairment and Alzheimer’s disease: a comparative overview. Mol Neurobiol.

[19] Hu WT, Holtzman DM, Fagan AM, Shaw LM, Perrin R (2012). Plasma multianalyte profiling in mild cognitive impairment and Alzheimer disease. Neurology.

[20] Johnstone D, Milward EA, Berretta R, Moscato P (2012). Multivariate protein signatures of pre-clinical Alzheimer’s disease in the Alzheimer’s disease neuroimaging initiative (ADNI) plasma proteome dataset. PLoS One.

[21] Magaki S, Mueller C, Dickson C, Kirsch W (2007). Increased production of inflammatory cytokines in mild cognitive impairment. Exp Gerontol.

[22] Ray S, Britschgi M, Herbert C, Takeda-Uchimura Y, Boxer A (2007). Classification and prediction of clinical Alzheimer’s diagnosis based on plasma signaling proteins. Nat Med.

[23] Galimberti D, Schoonenboom N, Scheltens P, Fenoglio C, Bouwman F (2006). Intrathecal chemokine synthesis in mild cognitive impairment and Alzheimer disease. Arch Neurol.

[24] Petersen RC (2004). Mild cognitive impairment as a diagnostic entity. J Intern Med.

[25] Zandi PP, Breitner JC (2001). Do NSAIDs prevent Alzheimer’s disease? And, if so, why? The epidemiological evidence. Neurobiol Aging.

[26] Rogers J, Kirby LC, Hempelman SR, Berry DL, McGeer PL (1993). Clinical trial of indomethacin in Alzheimer’s disease. Neurology.

[27] Zheng C, Zhou XW, Wang JZ (2016). The dual roles of cytokines in Alzheimer’s disease: update on interleukins, TNF-α, TGF-β and IFN-γ. Transl Neurodegener.

[28] Wang J, Tan L, Wang HF, Tan CC, Meng XF (2015). Anti-inflammatory drugs and risk of Alzheimer’s disease: an updated systematic review and meta-analysis. J Alzheimer’s Dis.

[29] ADAPT-FS Research Group (2015). Follow-up evaluation of cognitive function in the randomized Alzheimer’s Disease Anti-inflammatory Prevention Trial (ADAPT) and its Follow-up Study (ADAPT-FS). Alzheimer’s Dement.

[30] Martin BK, Szekely C, Brandt J, Piantadosi S, ADAPT Research Group (2008). Cognitive function over time in the Alzheimer’s Disease Anti-inflammatory Prevention Trial (ADAPT): results of a randomized, controlled trial of naproxen and celecoxib. Arch Neurol.

[31] Lyketsos CG, Breitner JC, Green RC, Martin BK, ADAPT Research Group (2007). Naproxen and celecoxib do not prevent AD in early results from a randomized controlled trial. Neurology.

[32] Vlad SC, Miller DR, Kowall NW, Felson DT (2008). Protective effects of NSAIDs on the development of Alzheimer disease. Neurology.

[33] Morimoto K, Horio J, Satoh H, Sue L, Beach T (2011). Expression profiles of cytokines in the brains of Alzheimer’s disease (AD) patients compared to the brains of non-demented patients with and without increasing AD pathology. J Alzheimer’s Dis.

[34] Mirra SS, Heyman A, McKeel D, Sumi SM, Crain BJ (1991). The Consortium to Establish a Registry for Alzheimer’s Disease (CERAD). Part II. Standardization of the neuropathologic assessment of Alzheimer’s disease. Neurology.

[35] Thal DR, Rüb U, Orantes M, Braak H (2002). Phases of A beta-deposition in the human brain and its relevance for the development of AD. Neurology.

[36] Braak H, Alafuzoff I, Arzberger T, Kretzschmar H, Del Tredici K (2006). Staging of Alzheimer disease-associated neurofibrillary pathology using paraffin sections and immunocytochemistry. Acta Neuropathol.

[37] Hyman BT, Trojanowski JQ (1997). Editorial on Consensus Recommendations for the Postmortem Diagnosis of Alzheimer’s Disease from the National Institute on Aging, and the Reagan Institute Working Group on Diagnostic Criteria for the Neuropathological Assessment of Alzheimer’s disease. J Neuropathol Exp Neurol.

[38] Lue LF, Kuo YM, Roher AE, Brachova L, Shen Y (1999). Soluble amyloid beta peptide concentration as a predictor of synaptic change in Alzheimer’s disease. Am J Pathol.

[39] Lue LF, Brachova L, Civin WH, Rogers J (1996). Inflammation, A beta deposition, and neurofibrillary tangle formation as correlates of Alzheimer’s disease neurodegeneration. J Neuropathol Exp Neurol.

[40] Serrano-Pozo A, Frosch MP, Masliah E, Hyman BT (2011). Neuropathological alterations in Alzheimer disease. Cold Spring Harb Perspect Med.

[41] Lowe J, Mirra SS, Hyman BT, Dickson DW, Lowe S, Louis D, Ellison DW (2008). Pathology of mild cognitive impairment and early Alzheimer’s disease, Alzheimer’s disease, Chapter 14. Ageing and dementia. Greenfield’ Neuropathology.

[42] Schneider JA, Arvanitakis Z, Leurgans SE, Bennett DA (2009). The neuropathology of probable Alzheimer disease and mild cognitive impairment. Ann Neurol.

[43] Dugger BN, Davis K, Malek-Ahmadi M, Hentz JG, Sandhu S (2015). Neuropathological comparisons of amnestic and nonamnestic mild cognitive impairment. BMC Neurol.

[44] Markesbery WR (2010). Neuropathologic alterations in mild cognitive impairment: a review. J Alzheimer’s Dis.

[45] Luterman JD, Haroutunian V, Yemul S, Ho L, Purohit D (2000). Cytokine gene expression as a function of the clinical progression of Alzheimer disease dementia. Arch Neurol.

[46] Lanzrein AS, Johnston CM, Petty VH, Jobst KA, King EM (1998). Longitudinal study of inflammatory factors in serum, cerebrospinal fluid, and brain tissue in Alzheimer disease: interleukin-1β, interleukin-6, interleukin-1 receptor antagonist, tumor necrosis factor-a, the soluble tumor necrosis factor receptors I and II, and a1-antichymotrypsin. Alzheimer Dis Assoc Disord.

[47] Araujo DM, Lapchak PA (1994). Induction of immune system mediators in the hippocampal formation in Alzheimer’s and Parkinson’s diseases: selective effects on specific interleukins and interleukin receptors. Neuroscience.

[48] Erten-Lyons D, Woltjer RL, Dodge H, Nixon R, Vorobik R (2009). Factors associated with resistance to dementia despite high Alzheimer disease pathology. Neurology.

[49] Knopman DS, Parisi JE, Salviati A, Floriach-Robert M, Boeve BF (2003). Neuropathology of cognitively normal elderly. J Neuropathol Exp Neurol.

[50] Esparza TJ, Zhao H, Cirrito JR, Cairns NJ, Bateman RJ (2013). Amyloid-beta oligomerization in Alzheimer dementia versus high-pathology controls. Ann Neurol.

[51] Perez-Nievas BG, Stein TD, Tai HC, Dols-Icardo O, Scotton TC (2013). Dissecting phenotypic traits linked to human resilience to Alzheimer’s pathology. Brain.

[52] Bjorklund NL, Reese LC, Sadagoparamanujam VM, Ghirardi V, Woltjer RL (2012). Absence of amyloid beta oligomers at the postsynapse and regulated synaptic Zn2+ in cognitively intact aged individuals with Alzheimer’s disease neuropathology. Mol Neurodegener.

[53] Maarouf CL, Daugs ID, Kokjohn TA, Walker DG, Hunter JM (2011). Alzheimer’s disease and non-demented high pathology control nonagenarians: comparing and contrasting the biochemistry of cognitively successful aging. PLoS ONE.

[54] Beach TG, Kuo YM, Spiegel K, Emmerling MR, Sue LI (2000). The cholinergic deficit coincides with Abeta deposition at the earliest histopathologic stages of Alzheimer disease. J Neuropathol Exp Neurol.

[55] Katzman R, Terry R, DeTeresa R, Brown T, Davies P (1988). Clinical, pathological, and neurochemical changes in dementia: a subgroup with preserved mental status and numerous neocortical plaques. Ann Neurol.

[56] Beach TG, Honer WG, Hughes LH (1997). Cholinergic fibre loss associated with diffuse plaques in the non-demented elderly: the preclinical stage of Alzheimer’s disease?. Acta Neuropathol.

[57] Caselli RJ, Walker D, Sue L, Sabbagh M, Beach T (2010). Amyloid load in nondemented brains correlates with APOE e4. Neurosci Lett.

[58] Wyss-Coray T, Rogers J (2012). Inflammation in Alzheimer disease-a brief review of the basic science and clinical literature. Cold Spring Harb Perspect Med.

[59] Basu A, Krady JK, Levison SW (2004). Interleukin-1: a master regulator of neuroinflammation. J Neurosci Res.

[60] Fu AK, Hung KW, Yuen MY, Zhou X, Mak DS (2016). IL-33 ameliorates Alzheimer’s disease-like pathology and cognitive decline. Proc Natl Acad Sci U S A.

[61] Liu L, Chan C (2014). The role of inflammasome in Alzheimer’s disease. Ageing Res Rev.

[62] Sutinen EM, Pirttilä T, Anderson G, Salminen A, Ojala JO (2012). Pro-inflammatory interleukin-18 increases Alzheimer’s disease-associated amyloid-β production in human neuron-like cells. J Neuroinflammation.

[63] Bossù P, Ciaramella A, Salani F, Vanni D, Palladino I (2010). Interleukin-18, from neuroinflammation to Alzheimer’s disease. Curr Pharm Des.

[64] Chapuis J, Hot D, Hansmannel F, Kerdraon O, Ferreira S (2009). Transcriptomic and genetic studies identify IL-33 as a candidate gene for Alzheimer’s disease. Mol Psychiatry.

[65] Craft JM, Watterson DM, Hirsch E, Van Eldik LJ (2005). Interleukin I receptor antagonist knockout mice show enhanced microglial activation and neuronal damage induced by intracerebroventricular infusion of human β-amyloid. J Neuroinflammation.

[66] Shaftel SS, Griffin WS, O’Banion MK (2008). The role of interleukin-1 in neuroinflammation and Alzheimer disease: an evolving perspective. J Neuroinflammation.

[67] Chakrabarty P, Li A, Ceballos-Diaz C, Eddy JA, Funk CC (2015). IL-10 alters immunoproteostasis in APP mice, increasing plaque burden and worsening cognitive behavior. Neuron.

[68] Guillot-Sestier MV, Doty KR, Gate D, Rodriguez J, Leung BP (2015). Il10 deficiency rebalances innate immunity to mitigate Alzheimerlike pathology. Neuron.

[69] Kawahara K, Suenobu M, Yoshida A, Koga K, Hyodo A (2012). Intracerebral microinjection of interleukin-4/interleukin-13 reduces β-amyloid accumulation in the ipsilateral side and improves cognitive deficits in young amyloid precursor protein 23 mice. Neuroscience.

[70] Szczepanik AM, Funes S, Petko W, Ringheim GE (2001). IL-4, IL-10 and IL-13 modulate Aβ (1–42)-induced cytokine and chemokine production in primary murine microglia and a human monocyte cell line. J Neuroimmunol.

[71] Khoshbakht T, Soosanabadi M, Neishaboury M, Kamali K, Karimlou M (2015). An Association Study on IL16 Gene Polymorphisms with the Risk of Sporadic Alzheimer’s Disease. Avicenna J Med Biotechnol.

[72] Di Rosa M, Dell’Ombra N, Zambito AM, Malaguarnera M, Nicoletti F (2006). Chitotriosidase and inflammatory mediator levels in Alzheimer’s disease and cerebrovascular dementia. Eur J Neurosci.

[73] Yun HM, Kim JA, Hwang CJ, Jin P, Baek MK (2015). Neuroinflammatory and Amyloidogenic Activities of IL-32β in Alzheimer’s Disease. Mol Neurobiol.

[74] Azizi G, Khannazer N, Mirshafiey A (2014). The Potential Role of Chemokines in Alzheimer’s Disease Pathogenesis. Am J Alzheimer’s Dis Other Demen.

[75] Streit WJ, Conde JR, Harrison JK (2001). Chemokines and Alzheimer’s disease. Neurobiol Aging.

[76] Bacher M, Deuster O, Aljabari B, Egensperger R, Neff F (2010). The role of macrophage migration inhibitory factor in Alzheimer’s disease. MolMed.

[77] Town T, Laouar Y, Pittenger C, Mori T, Szekely CA (2008). Blocking TGF-beta-Smad2/3 innate immune signaling mitigates Alzheimer-like pathology. Nat Med.

[78] Tesseur I, Zou K, Esposito L, Bard F, Berber E (2006). Deficiency in neuronal TGF-beta signaling promotes neurodegeneration and Alzheimer’s pathology. J Clin Invest.

[79] Beach TG, Sue LI, Walker DG, Roher AE, Lue LF (2008). The Sun Health Research Institute brain donation program: description and experience, 1987–2007. Cell Tissue Bank.

[80] Fein JA, Sokolow S, Miller CA, Vinters HV, Yang F (2008). Co-localization of amyloid beta and tau pathology in Alzheimer’s disease synaptosomes. Am J Pathol.

[81] Fukumoto H, Rosene DL, Moss MB, Raju S, Hyman BT (2004). Beta-secretase activity increases with aging in human, monkey, and mouse brain. Am J Pathol.

[82] Ikonomovic MD, Klunk WE, Abrahamson EE, Mathis CA, Price JC (2008). Post-mortem correlates of in vivo PiB-PET amyloid imaging in a typical case of Alzheimer’s disease. Brain.

[83] Mann DM, Iwatsubo T, Snowden JS (1996). Atypical amyloid (A beta) deposition in the cerebellum in Alzheimer’s disease: an immunohistochemical study using end-specific A beta monoclonal antibodies. Acta Neuropathologica.

[84] Mann DM, Jones D, Prinja D, Purkiss MS (1990). The prevalence of amyloid (A4) protein deposits within the cerebral and cerebellar cortex in Down’s syndrome and Alzheimer’s disease. Acta Neuropathol.

[85] Joachim CL, Morris JH, Selkoe DJ (1989). Diffuse senile plaques occur commonly in the cerebellum in Alzheimer’s disease. Am J Pathol.

[86] Du J, Sun B, Chen K, Fan L, Wang Z (2009). Antagonist of peroxisome proliferator-activated receptor gamma induces cerebellar amyloid-β levels and motor dysfunction in APP/PS1 transgenic mice. Biochem Biophys Res Commun.

[87] Phares TW, Kean RB, Mikheeva T, Hooper DC (2006). Regional differences in blood-brain barrier permeability changes and inflammation in the apathogenic clearance of virus from the central nervous system. J Immunol.

[88] Carriba P, Jimenez S, Navarro V, Moreno-Gonzalez I, Barneda-Zahonero B (2015). Amyloid-β reduces the expression of neuronal FAIM-L, thereby shifting the inflammatory response mediated by TNFa from neuronal protection to death. Cell Death Dis.

[89] Ojala J, Alafuzoff I, Herukka SK, van Groen T, Tanila H (2009). Expression of interleukin-18 is increased in the brains of Alzheimer’s disease patients. Neurobiol Aging.

[90] Wood JA, Wood PL, Ryan R, Graff-Radford NR, Pilapil C (1993). Cytokine indices in Alzheimer’s temporal cortex: no changes in mature IL-1β or IL-1RA but increases in the associated acute phase proteins IL-6, a2-macroglobulin and C-reactive protein. Brain Res.

[91] Konishi Y, Beach T, Sue LI, Hampel H, Lindholm K (2003). The temporal localization of frame-shift ubiquitin-B and amyloid precursor protein, and complement proteins in the brain of non-demented control patients with increasing Alzheimer’s disease pathology. Neurosci Lett.

[92] Singhrao SK, Neal JW, Rushmere NK, Morgan BP, Gasque P (1999). Differential expression of individual complement regulators in the brain and choroid plexus. Lab Invest.

[93] Vedeler C, Ulvestad E, Bjørge L, Conti G, Williams K (1994). The expression of CD59 in normal human nervous tissue. Immunology.

[94] Yang LB, Li R, Meri S, Rogers J, Shen Y (2000). Deficiency of complement defense protein CD59 may contribute to neurodegeneration in Alzheimer’s disease. J Neurosci.

[95] Shen Y, Halperin JA, Lee CM (1995). Complement-mediated neurotoxicity is regulated by homologous restriction. Brain Res.

[96] Yasojima K, McGeer EG, McGeer PL (1999). Complement regulators C1 inhibitor and CD59 do not significantly inhibit complement activation in Alzheimer disease. Brain Res.

[97] Vasconcelos AR, Cabral-Costa JV, Mazucanti CH, Scavone C, Kawamoto EM (2016). The Role of Steroid Hormones in the Modulation of Neuroinflammation by Dietary Interventions. Front Endocrinol (Lausanne).

[98] Landfield PW, Blalock EM, Chen KC, Porter NM (2007). A new glucocorticoid hypothesis of brain aging: implications for Alzheimer’s disease. Curr Alzheimer Res.

[99] Sorrells SF, Munhoz CD, Manley NC, Yen S, Sapolsky RM (2014). Glucocorticoids increase excitotoxic injury and inflammation in the hippocampus of adult male rats. Neuroendocrinology.

[100] Munhoz CD, Sorrells SF, Caso JR, Scavone C, Sapolsky RM (2010). Glucocorticoids exacerbate lipopolysaccharide-induced signaling in the frontal cortex and hippocampus in a dose-dependent manner. J Neurosci.

[101] de Pablos RM, Villarán RF, Argüelles S, Herrera AJ, Venero JL (2006). Stress increases vulnerability to inflammation in the rat prefrontal cortex. J Neurosci.

[102] Sorrells SF, Caso JR, Munhoz CD, Sapolsky RM (2009). The stressed CNS: when glucocorticoids aggravate inflammation. Neuron.

[103] Dinkel K, MacPherson A, Sapolsky RM (2003). Novel glucocorticoid effects on acute inflammation in the CNS. J Neurochem.

[104] Aisen PS, Davis KL, Berg JD, Schafer K, Campbell K (2000). A randomized controlled trial of prednisone in Alzheimer’s disease. Alzheimer’s Disease Cooperative Study. Neurology.

[105] The ADAPT Research Group (2013). Results of a follow-up study to the randomized Alzheimer’s Disease Anti-inflammatory Prevention Trial (ADAPT). Alzheimer’s Dement.

[106] Breitner JC, Baker LD, Montine TJ, Meinert CL, Lyketsos CG (2011). Extended results of the Alzheimer’s disease anti-inflammatory prevention trial. Alzheimer’s Dement.

[107] Kukar T, Prescott S, Eriksen JL, Holloway V, Murphy MP (2007). Chronic administration of R-flurbiprofen attenuates learning impairments in transgenic amyloid precursor protein mice. BMC Neurosci.

[108] Sung S, Yang H, Uryu K, Lee EB, Zhao L (2004). Modulation of nuclear factor-kappa B activity by indomethacin influences A beta levels but not A beta precursor protein metabolism in a model of Alzheimer’s disease. Am J Pathol.

[109] Lim GP, Yang F, Chu T, Gahtan E, Ubeda O (2001). Ibuprofen effects on Alzheimer pathology and open field activity in APPsw transgenic mice. Neurobiol Aging.

[110] Lim GP, Yang F, Chu T, Chen P, Beech W (2000). Ibuprofen suppresses plaque pathology and inflammation in a mouse model for Alzheimer’s disease. J Neurosci.

[111] Wang J, Tan L, Wang HF, Tan CC, Meng XF (2015). Anti-inflammatory drugs and risk of Alzheimer’s disease: an updated systematic review and meta-analysis. J Alzheimer’s Dis.

[112] Sastre M, Dewachter I, Rossner S, Bogdanovic N, Rosen E (2006). Nonsteroidal anti-inflammatory drugs repress beta-secretase gene promoter activity by the activation of PPARgamma. Proc Natl Acad Sci U S A.

[113] Weggen S, Eriksen JL, Das P, Sagi SA, Wang R (2001). A subset of NSAIDs lower amyloidogenic Abeta42 independently of cyclooxygenase activity. Nature.

[114] Sonnen JA, Larson EB, Walker RL, Haneuse S, Crane PK (2010). Nonsteroidal anti-inflammatory drugs are associated with increased neuritic plaques. Neurology.

[115] Kukal T, Murphy MP, Eriksen JL, Sagi SA, Weggen S (2005). Diverse compounds mimic Alzheimer disease-causing mutations by augmenting αβ42 production. Nat Med.

[116] Cole GM, Frautschy SA (2010). Mechanisms of action of non-steroidal anti-inflammatory drugs for the prevention of Alzheimer’s disease. CNS Neurol Disord Drug Targets.

[117] Aisen PS, Thal LJ, Ferris SH, Assaid C, Nessly ML (2008). Rofecoxib in patients with mild cognitive impairment: further analyses of data from a randomized, double-blind, trial. Curr Alzheimer Res.

[118] Small GW, Siddarth P, Silverman DHS, Ercoli LM, Miller KJ (2008). Cognitive and cerebral metabolic effects of celecoxib versus placebo in people with age-related memory loss: randomized controlled study. Am J Geriatr Psychiatry.

[119] Frautschy SA (2010). Thinking outside the box about COX-1 in Alzheimer’s disease. Neurobiol Dis.

[120] Gómez-Isla T, Blesa R, Boada M, Clarimón J, Del Ser T (2008). A randomized, double-blind, placebo controlled-trial of triflusal in mild cognitive impairment: the TRIMCI study. Alzheimer Dis Assoc Disord.

[121] Kotilinek LA, Westerman MA, Wang Q, Panizzon K, Lim GP (2008). Cyclooxygenase-2 inhibition improves amyloid-beta-mediated suppression of memory and synaptic plasticity. Brain.

[122] Moon JH (2016). Endocrine Risk Factors for Cognitive Impairment. Endocrinol Metab (Seoul).

[123] De Felice FG, Lourenco MV (2015). Brain metabolic stress and neuroinflammation at the basis of cognitive impairment in Alzheimer’s disease. Front Aging Neurosci.

[124] De Felice FG, Ferreira ST (2014). Inflammation, defective insulin signaling, and mitochondrial dysfunction as common molecular denominators connecting type 2 diabetes to Alzheimer’s disease. Diabetes.

[125] Ferreira ST, Clarke JR, Bomfim TR, De Felice FG (2014). Inflammation, defective insulin signaling, and neuronal dysfunction in Alzheimer’s disease. Alzheimer’s Dement.

[126] Barrientos RM, Frank MG, Watkins LR, Maier SF (2010). Memory impairments in healthy aging: Role of aging-induced microglial sensitization. Aging Dis.

[127] Romeo GR, Lee J, Shoelson SE (2012). Metabolic syndrome, insulin resistance, and roles of inflammation - Mechanisms and therapeutic targets. Arterioscler Thromb Vasc Biol.

[128] Bomfim TR, Forny-Germano L, Sathler LB, Brito-Moreira J, Houzel J-C (2012). An anti-diabetes agent protects the mouse brain from defective insulin signaling caused by Alzheimer’s disease-associated αβ oligomers. J Clin Invest.

[129] Yarchoan M, Arnold SE (2014). Repurposing diabetes drugs for brain insulin resistance in Alzheimer disease. Diabetes.

[130] de la Monte SM (2012). Contributions of brain insulin resistance and deficiency in amyloid-related neurodegeneration in Alzheimer’s disease. Drugs.

[131] Chiu S-L, Chen CM, Cline HT (2008). Insulin receptor signaling regulates synapse number, dendritic plasticity, and circuit function in vivo. Neuron.

[132] Dou J-T, Chen M, Dufour F, Alkon DL, Zhao W-Q (2005). Insulin receptor signaling in long-term memory consolidation following spatial learning. Learn Mem.

[133] McClean PL, Parthsarathy V, Faivre E, Hölscher C (2011). The diabetes drug liraglutide prevents degenerative processes in a mouse model of Alzheimer’s disease. J Neurosci.

[134] Lourenco MV, Clarke JR, Frozza RL, Bomfim TR, Forny-Germano L (2013). TNF-α mediates PKR-dependent memory impairment and brain IRS-1 inhibition induced by Alzheimer’s β-amyloid oligomers in mice and monkeys. Cell Metab.

[135] Kornelius E, Lin C-L, Chang H-H, Li H-H, Huang W-N (2015). DPP-4 inhibitor linagliptin attenuates αβ-induced cytotoxicity through activation of AMPK in neuronal cells. CNS Neurosci Ther.

[136] Claxton A, Baker LD, Hanson A, Trittschuh EH, Cholerton B (2015). Long-acting intranasal insulin detemir improves cognition for adults with mild cognitive impairment or early-stage Alzheimer’s disease dementia. J Alzheimer’s Dis.

[137] Talbot K, Wang H-Y, Kazi H, Han L-Y, Bakshi KP (2012). Demonstrated brain insulin resistance in Alzheimer’s disease patients is associated with IGF-1 resistance, IRS-1 dysfunction, and cognitive decline. J Clin Invest.

[138] Pandey MK, DeGrado TR (2016). Glycogen Synthase Kinase-3 (GSK-3)-Targeted Therapy and Imaging. Theranostics.

[139] Engel T, Hernández F, Avila J, Lucas JJ (2006). Full reversal of Alzheimer’s disease-like phenotype in a mouse model with conditional overexpression of glycogen synthase kinase-3. J Neurosci.

[140] McGeer EG, McGeer PL (2010). Neuroinflammation in Alzheimer’s disease and mild cognitive impairment: a field in its infancy. J Alzheimer’s Dis.

[141] Tobinick E (2007). Perispinal etanercept for treatment of Alzheimer’s disease. Curr Alzheimer Res.

[142] Perry RT, Collins JS, Wiener H, Acton R, Go RC (2001). The role of TNF and its receptors in Alzheimer’s disease. Neurobiol Aging.

[143] Janelsins MC, Mastrangelo MA, Park KM, Sudol KL, Narrow WC (2008). Chronic neuron-specific tumor necrosis factor-a expression enhances the local inflammatory environment ultimately leading to neuronal death in 3xTG-AD mice. Am J Pathol.

[144] White JA, Manelli AM, Holmberg KH, Van Eldik LJ, Ladu MJ (2005). Differential effects of oligomeric and fibrillar amyloid-beta 1–42 on astrocyte-mediated inflammation. Neurobiol Dis.

[145] Combs CK, Karlo JC, Kao SC, Landreth GE (2001). β-Amyloid stimulation of microglia and monocytes results in TNFa-dependent expression of inducible nitric oxide synthase and neuronal apoptosis. J Neurosci.

[146] Park KM, Bowers WJ (2010). Tumor necrosis factor-alpha mediated signaling in neuronal homeostasis and dysfunction. Cell Signal.

[147] Tobinick E (2009). Tumour necrosis factor modulation for treatment of Alzheimer’s disease: rationale and current evidence. CNS Drugs.

[148] Leonoudakis D, Zhao P, Beattie EC (2008). Rapid tumor necrosis factor a-induced exocytosis of glutamate receptor 2-lacking AMPA receptors to extrasynaptic plasma membrane potentiates excitotoxicity. J Neurosci.

[149] Medeiros R, Prediger RD, Passos GF, Pandolfo P, Duarte FS (2007). Connecting TNF-alpha signaling pathways to iNOS expression in a mouse model of Alzheimer’s disease: relevance for the behavioral and synaptic deficits induced by amyloid beta protein. J Neurosci.

[150] He P, Zhong Z, Lindholm K, Berning L, Lee W (2007). Deletion of tumor necrosis factor death receptor inhibits amyloid β generation and prevents learning and memory deficits in Alzheimer’s mice. J Cell Biol.

[151] McAlpine FE, Lee JK, Harms AS, Ruhn KA, Blurton-Jones M (2009). Inhibition of soluble TNF signaling in a mouse model of Alzheimer’s disease prevents pre-plaque amyloid-associated neuropathology. Neurobiol Dis.

[152] Chakrabarty P, Herring A, Ceballos-Diaz C, Das P, Golde TE (2011). Hippocampal expression of murine TNFa results in attenuation of amyloid deposition in vivo. Mol Neurodegener.

[153] Saha RN, Ghosh A, Palencia CA, Fung YK, Dudek SM (2009). TNF-alpha preconditioning protects neurons via neuron-specific up-regulation of CREB-binding protein. J Immunol.

[154] Orellana DI, Quintanilla RA, Maccioni RB (2007). Neuroprotective effect of TNFalpha against the beta-amyloid neurotoxicity mediated by CDK5 kinase. Biochim Biophys Acta.

[155] Barger SW, Hörster D, Furukawa K, Goodman Y, Krieglstein J (1995). Tumor necrosis factors a and β protect neurons against amyloid β-peptide toxicity: evidence for involvement of a κB-binding factor and attenuation of peroxide and Ca2+ accumulation. Proc Natl Acad Sci USA.

[156] Dolga AM, Granic I, Blank T, Knaus HG, Spiess J (2008). TNF-α-mediates neuroprotection against glutamate-induced excitotoxicity via NF-κB-dependent up-regulation of KCa2.2 channels. J Neurochem.

[157] Cheng B, Christakos S, Mattson MP (1994). Tumor necrosis factors protect neurons against metabolic-excitotoxic insults and promote maintenance of calcium homeostasis. Neuron.

[158] Patel JR, Brewer GJ (2008). Age-related changes to tumor necrosis factor receptors affect neuron survival in the presence of beta-amyloid. J Neurosci Res.

[159] Vandenabeele P, Declercq W, Beyaert R, Fiers W (1995). Two tumour necrosis factor receptors: structure and function. Trends Cell Biol.

[160] Faustman D, Davis M (2010). TNF receptor 2 pathway: drug target for autoimmune diseases. Nat Rev Drug Discov.

[161] Wajant H, Pfizenmaier K, Scheurich P (2003). Tumor necrosis factor signaling. Cell Death Differ.

[162] McCoy MK, Tansey MG (2008). TNF signaling inhibition in the CNS: implications for normal brain function and neurodegenerative disease. J Neuroinflammation.

[163] Montgomery SL, Narrow WC, Mastrangelo MA, Olschowka JA, O’Banion MK (2013). Chronic neuron-and age-selective down-regulation of TNF receptor expression in triple-transgenic Alzheimer disease mice leads to significant modulation of amyloid- and tau-related pathologies. Am J Pathol.

[164] Pimentel-Muiños FX, Seed B (1999). Regulated commitment of TNF receptor signaling: a molecular switch for death or activation. Immunity.

[165] Pickering M, Cumiskey D, O’Connor JJ (2005). Actions of TNF-alpha on glutamatergic synaptic transmission in the central nervous system. Exp Physiol.

[166] Marchetti L, Klein M, Schlett K, Pfizenmaier K, Eisel ULM (2004). Tumor necrosis factor (TNF)-mediated neuroprotection against glutamate-induced excitotoxicity is enhanced by N-methyl-D-aspartate receptor activation. Essential role of a TNF receptor 2-mediated phosphatidylinositol 3-kinase-dependent NF-kappa B pathway. J Biol Chem.

[167] Saleem M, Herrmann N, Swardfager W, Eisen R, Lanctot KL (2015). Inflammatory Markers in Mild Cognitive Impairment: A Meta-Analysis. J Alzheimer’s Dis.

[168] Huberman M, Shalit F, Roth-Deri I, Gutman B, Brodie C (1994). Correlation of cytokine secretion by mononuclear cells of Alzheimer patients and their disease stage. J Neuroimmunol.

[169] Sharma V, Thakur V, Singh SN, Guleria R (2012). Tumor necrosis factor and Alzheimer’s disease: a cause and consequence relationship. Klinik Psikofarmakoloji Bülteni (Bull Clin Psychopharmacol).

[170] Buxbaum JD, Liu KN, Luo Y, Slack JL, Stocking KL (1998). Evidence that tumor necrosis factor a converting enzyme is involved in regulated a-secretase cleavage of the Alzheimer amyloid protein precursor. J Biol Chem.

[171] Reddy P, Slack JL, Davis R, Cerretti DP, Kozlosky CJ (2000). Functional analysis of the domain structure of tumor necrosis factor-alpha converting enzyme. J Biol Chem.

[172] Jiang H, Hampel H, Prvulovic D, Wallin A, Blennow K (2011). Elevated CSF levels of TACE activity and soluble TNF receptors in subjects with mild cognitive impairment and patients with Alzheimer’s disease. Mol Neurodegener.

[173] Kim ML, Zhang B, Mills IP, Milla ME, Brunden KR (2008). Effects of TNFalpha-converting enzyme inhibition on amyloid beta production and APP processing in vitro and in vivo. J Neurosci.

[174] Wang H, Czura CJ, Tracey KJ, Thomson AW, Lotze MT (2003). Tumor necrosis factor. The Cytokine Handbook.

[175] Marques-Fernandez F, Planells-Ferrer L, Gozzelino R, Galenkamp KM, Reix S (2013). TNFα induces survival through the FLIP-L-dependent activation of the MAPK/ERK pathway. Cell Death Dis.

[176] Micheau O, Tschopp J (2003). Induction of TNF receptor I-mediated apoptosis via two sequential signaling complexes. Cell.

[177] Del Villar K, Miller CA (2004). Down-regulation of DENN/MADD, a TNF receptor binding protein, correlates with neuronal cell death in Alzheimer’s disease brain and hippocampal neurons. Proc Natl Acad Sci USA.

[178] Varfolomeev EE, Ashkenazi A (2004). Tumor necrosis factor: an apoptosis JuNKie?. Cell.

[179] Fontaine V, Mohand-Said S, Hanoteau N, Fuchs C, Pfizenmaier K (2002). Neurodegenerative and neuroprotective effects of tumor Necrosis factor (TNF) in retinal ischemia: opposite roles of TNF receptor 1 and TNF receptor 2. J Neurosci.

[180] Bruce AJ, Boling W, Kindy MS, Peschon J, Kraemer PJ (1996). Altered neuronal and microglial responses to excitotoxic and ischemic brain injury in mice lacking TNF receptors. Nat Med.

[181] Montgomery SL, Mastrangelo MA, Habib D, Narrow WC, Knowlden SA (2011). Ablation of TNF-RI/RII expression in Alzheimer’s disease mice leads to an unexpected enhancement of pathology: implications for chronic pan-TNF-α suppressive therapeutic strategies in the brain. Am J Pathol.

[182] Jiang H, He P, Xie J, Staufenbiel M, Li R (2014). Genetic deletion of TNFRII gene enhances the Alzheimer-like pathology in an APP transgenic mouse model via reduction of phosphorylated IκBα. Hum Mol Genet.

[183] Cheng X, Yang L, He P, Li R, Shen Y (2010). Differential activation of tumor necrosis factor receptors distinguishes between brains from Alzheimer’s disease and non-demented patients. J Alzheimer’s Dis.

[184] Culpan D, Cornish A, Love S, Kehoe PG, Wilcock GK (2007). Protein and gene expression of tumour necrosis factor receptors I and II and their promoter gene polymorphisms in Alzheimer’s disease. Exp Gerontol.

[185] González IL, Garcia-Esparcia P, Llorens F, Ferrer I (2016). Genetic and transcriptomic profiles of inflammation in neurodegenerative diseases: Alzheimer, Parkinson, Creutzfeldt-Jacob and tauopathies. Int J Mol Sci.

[186] Shi JQ, Wang BR, Jiang WW, Chen J, Zhu YW (2011). Cognitive improvement with intrathecal administration of infliximab in a woman with Alzheimer’s disease. J Am Geriatr Soc.

[187] Tobinick EL (2010). Perispinal etanercept: a new therapeutic paradigm in neurology. Expert Rev Neurother.

[188] Tobinick EL, Gross H (2008). Rapid improvement in verbal fluency and aphasia following perispinal etanercept in Alzheimer’s disease. BMC Neurol.

[189] Tobinick EL, Gross H (2008). Rapid cognitive improvement in Alzheimer’s disease following perispinal etanercept administration. J Neuroinflammation.

[190] Butchart J, Brook L, Hopkins V, Teeling J, Püntener U (2015). Etanercept in Alzheimer disease: A randomized, placebo-controlled, double-blind, phase 2 trial. Neurology.

[191] He P, Cheng X, Staufenbiel M, Li R, Shen Y (2013). Long-term treatment of thalidomide ameliorates amyloid-like pathology through inhibition of β-secretase in a mouse model of Alzheimer’s disease. PLoS ONE.

[192] Greig NH, Giordano T, Zhu X, Yu QS, Perry TA (2004). Thalidomide-based TNF-alpha inhibitors for neurodegenerative diseases. Acta Neurobiol Exp (Wars).

[193] Ferretti MT, Allard S, Partridge V, Ducatenzeiler A, Cuello AC (2012). Minocycline corrects early, pre-plaque neuroinflammation and inhibits BACE-1 in a transgenic model of Alzheimer’s disease-like amyloid pathology. J Neuroinflammation.

[194] Fischer R, Kontermann RE, Maier O (2015). Targeting sTNF/TNFR1 signaling as a new therapeutic strategy. Antibodies.

[195] Williams SK, Maier O, Fischer R, Fairless R, Hochmeister S (2014). Antibody-mediated inhibition of TNFR1 attenuates disease in a mouse model of multiple sclerosis. PLoS One.

[196] Dansokho C, Ait Ahmed D, Aid S, Toly-Ndour C, Chaigneau T (2016). Regulatory T cells delay disease progression in Alzheimer-like pathology. Brain.

